# Integration of the PubAnnotation ecosystem in the development of a web-based search tool for alternative methods

**DOI:** 10.5808/GI.2020.18.2.e18

**Published:** 2020-06-15

**Authors:** Mariana Neves

**Affiliations:** German Federal Institute for Risk Assessment (BfR), German Centre for the Protection of Laboratory Animals (Bf3R), 12277 Berlin, Germany

**Keywords:** animal testing alternatives, information storage and retrieval, text mining

## Abstract

Finding publications that propose alternative methods to animal experiments is an important but time-consuming task since researchers need to perform various queries to literature databases and screen many articles to assess two important aspects: the relevance of the article to the research question, and whether the article’s proposed approach qualifies to being an alternative method. We are currently developing a Web application to support finding alternative methods to animal experiments. The current (under development) version of the application utilizes external tools and resources for document processing, and relies on the PubAnnotation ecosystem for annotation querying, annotation storage, dictionary-based tagging of cell lines, and annotation visualization. Currently, our two PubAnnotation repositories for discourse elements contain annotations for more than 110k PubMed documents. Further, we created an annotator for cell lines that contain more than 196k terms from Cellosaurus. Finally, we are experimenting with TextAE for annotation visualization and for user feedback.

## Introduction

According to the Directive 2010/63/EU (https://eur-lex.europa.eu/legal-content/EN/ALL/?uri=CELEX%3A32010L0063), researchers who plan to carry out animal experimentation are required to examine whether alternative methods not entailing the use of a live animal are already available for the planned research purpose (replacement). In addition, the chosen method should ensure that the animal number is reduced to a minimum without comprising the objective (reduction), and to reduce the possible pain, distress, and suffering (refinement). These measures are known as the 3R principles.

When searching for alternative methods to animal experiments, researchers have to carry out various queries to bibliographic databases, e.g., PubMed (https://www.ncbi.nlm.nih.gov/pubmed/), and carefully analyze the potential candidate articles. For each of these potential articles, the researcher should check whether it addresses two important issues: (1) a method for replacement, and (2) the planned research question. To assist researchers in their search for alternative methods, we are currently developing a Web application that addresses these two aspects. We rank the potential candidate articles based on the similarity of the research question (with regard to an input article) and identify the proposed methods in each of the articles.

For the implementation of the Web application, we rely on various tools, such as document classification, named-entity recognition, and annotation storage, among others. In the scope of the BLAH6 Hackathon (https://blah6.linkedannotation.org/), we integrated the PubAnnotation ecosystem [1] into the backend of our application. PubAnnotation contains three main components that can support our application for some of those tasks: the PubAnnotation repository, PubDictionaries, and the TextAE annotation tool.

Here we describe the integration of these tools into our application. We start by introducing our application, followed by how the components are being integrated into it. The Web application is still under development and not yet available for the final user. However, the resources that we created in the PubAnnotation platform are already available to the research community.

## The Web Application

**Fig. 1.**
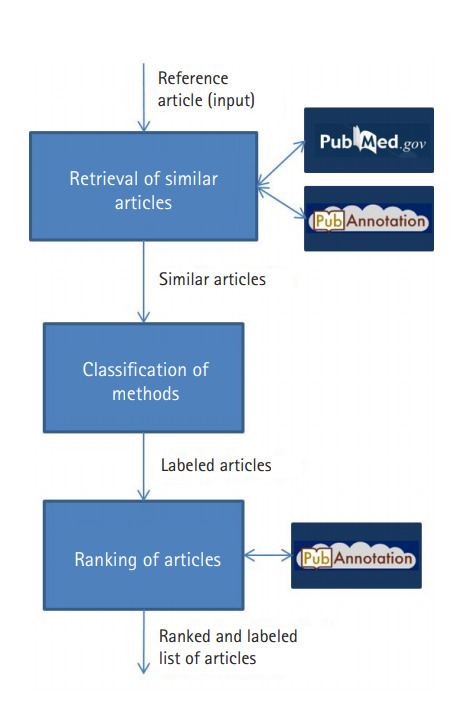
Workflow of the interaction between the application with PubMed and PubAnnotation.

Fig. 1 shows an overview of the real-time interaction of the Web application with PubMed and PubAnnotation. Given a reference article as input, our application retrieves the so-called similar articles from PubMed (https://www.ncbi.nlm.nih.gov/pubmed), i.e., the ones that were pre-compiled by PubMed [2]. The tool performs two processing tasks based on the title and abstracts of these similar articles: (1) classification of the proposed methods; and (2) ranking of the retrieved similar articles according to the similarity of their research questions to the one in the reference article.

The classification of the proposed methods will utilize machine learning algorithms to be trained based on manually annotated articles (abstracts), which are currently being manually labeled. These labels cover the various types of methods that are relevant for our domain, such as whether the experiments have been carried out *in vivo* (e.g., vertebrates, invertebrates) or *in vitro* (e.g., cell lines or organs). Further, we are also experimenting with named-entity recognition tools to support this task. We focus on entities which are still not well supported by the existing tools, e.g., cell lines, and on a dictionary-based approach that relies on the comprehensive Cellosaurus resource [3].

For the ranking task, the application calculates the text similarity between the reference article and each of the PubMed similar articles, and the resulting scores are used to rank these articles. For the text similarity, we utilize the TextFlow tool [4]. However, instead of relying on the whole abstract of the articles, we utilize only the most relevant discourse categories (or zones), such as “introduction” and “results”. This is due to the fact that only some parts of the abstract potentially describe the research question.

We recently published a study in which we compared four tools for the extraction of the zones and evaluated them on seven use studies (https://github.com/mariananeves/scientific-elements-text-similarity) [5]. Our study also demonstrated that using pre-selected zones, instead of the whole abstracts, yields better performance in the ranking task.

The zones can be manually annotated or automatically detected. The manually annotated zones are the original ones included in the structured abstracts in Pubmed (https://www.nlm.nih.gov/bsd/policy/structured_abstracts.html). However, given that not all abstracts in PubMed are structured, we automatically extract the zones for the remaining articles using the ArguminSci tool [6]. This was the best performing tool according to our study [5].

Finally, our Web application will contain visual components to display the abstract of the articles involved in the search. We currently consider two scenarios. The first is the visualization of the reference article in order to obtain feedback from the user, e.g., the research question in mind, by asking the user to highlight this information on the text. The second scenario is a side-by-side display of the reference article and each one of the retrieved similar articles in order to compare two articles and further gather user feedback.

## Integration with the PubAnnotation Ecosystem

We are currently integrating the PubAnnotaton ecosystem in various components of our Web application in order to support various tasks, namely, storage, alignment, named-entity recognition, and visualization of annotations. Here we describe how each of the tools is being integrated in our application.

### PubAnnotation database

We utilize the PubAnnotatin database to allow easy storage and retrieval of PubMed titles, abstracts, and their annotations. We store the zones coming from the reference article and its similar articles into one of the two repositories that we created in PubAnnotation, depending on the origin of these zones: (1) the PubMed_Structured_Abstracts repository (http://pubannotation.org/projects/PubMed_Structured_Abstracts) for the original zones available in the structured abstracts in PubMed; and (2) the PubMed_ArguminSci repository (http://pubannotation.org/projects/PubMed_ArguminSci) for the zones automatically extracted by the ArguminSci tool [6]. Both repositories are public and the annotations (zones) can be retrieved using the PubAnnotation API (http://www.pubannotation.org/docs/intro/).

**Fig. 2.**
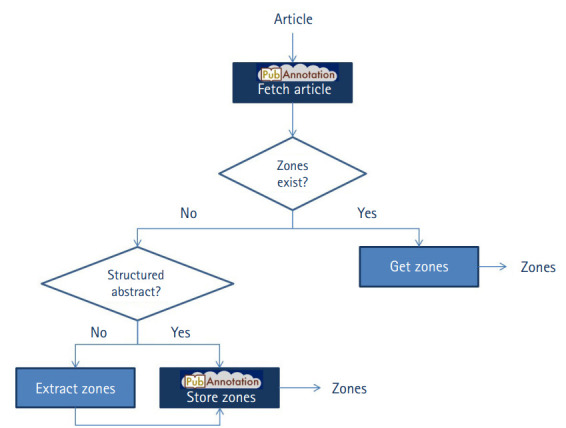
Workflow of the interaction of the Web application with the PubAnnotation API. The components that perform calls to API are identified with the logo of PubAnnotation.

For each article processed by our application, both reference articles or similar articles, we first check whether the article is already included in the PubAnnotation (cf. “fetch article” in Fig. 2), i.e., in any of its repositories. The output is either a message that the article is inexistent or a JSON object that includes the article’s title, abstract, and its annotations, which may come from various repositories in PubAnnotaton. We check whether annotations already exist from any of our two repositories described above (cf. “get zones” in Fig. 2). If any zones could be found, these are returned to be further processed by the Web application.

In case that no zones have been stored for the article in none of our two repositories, we first check whether the article contains a structured abstract. This information is contained in the data retrieved from PubMed. If the article contains a structured abstract, its zones are simply stored into PubAnnotation (cf. “store zones” in Fig. 2) and will be available for future queries. Otherwise, we extract the zones using the ArguminSci tool, followed by their storage into PubAnnotation. For any of the two situations, we store the zones in PubAnnotation in a two-steps procedure: (1) we add the article into the corresponding repository (either PubMed_Structured_Abstracts or PubMed_ArguminSci), and (2) we add the annotations into the same repository. It is not possible to perform the second step if the article was not previously included in the repository.

As of May 2020, the PubMed_Structured_Abstracts repository contains more than 31k documents while the PubMed_ArguminSci repository holds almost 80k documents. Therefore, we can state that less than 30% of the documents processed by our application included a structured abstract, while we had to perform predictions for zones for more than 70% of them. These documents consist of reference articles and the corresponding similar articles derived from the various queries that we made to our application in the last months, but also from the machine learning experiments that we carried out for the document classification step. Currently, we do not plan to include zoning annotations for all articles in PubMed, but just for those that happen to be processed by our application during our various experiments, and later, from the queries made by the users. Therefore, the repositories should incrementally grow with the time.

Another interesting feature in PubAnnotation is the annotation alignment. We deal with annotations retrieved from two sources, i.e., ArguminSci and PubMed Structured Abstracts, whose annotations might have been derived from a slightly different version of the article’s abstract, or the corresponding text somehow altered by the tool during processing. The annotation alignment function in PubAnnotation automatically converts the offsets of these annotations to the article’s abstract that is stored in PubAnnotation. Therefore, this function relieves us from writing customized scripts for dealing with the annotations returned by the various resources or tools.

Currently, no storage in PubAnnotation is being carried out for annotations coming from the classification task. This is due to a couple of reasons. First, the performance of our algorithms is not yet satisfactory. Further, document-level annotations are currently not supported by the JSON format of PubAnnotation. However, we plan to store them in PubAnnotation in the near future.

### TextAE (Text Annotation Editor)

Besides using the PubAnnotation ecosystem for annotation storage, we also plan to rely on other tools of the plattform in our Web application. For instance, we are currently experimenting with the TextAE tool (http://textae.pubannotation.org/) for displaying articles and annotations to the user. TextAE can be embedded into a HTML page to display the text and annotations that are passed in the JSON format. TextAE can be used in both of our visualization scenarios, i.e., either for displaying single articles or a side-by-side comparison. For the first scenario, an editable version of TextAE can potentially be used for collecting user feedback on the reference article, i.e., through text highlighting. For the second scenario (side-by-side comparison), we currently display annotations for species, disease, and chemicals from PubTator Central [7] using its annotator (http://pubannotation.org/annotators/PubTator) that is currently available in PubAnnotation. We also envisage relying on a side-by-side comparison to gather feedback from the user about the similarity of both research questions.

### PubDictionaries

We are also experimenting with PubDictionaries in the PubAnnotation ecosystem. Given a dictionary composed of terms (i.e., sets of identifiers and names), it is possible to perform a dictionary-based named-entity recognition by matching the terms in the dictionary to the title and abstract of articles in PubAnnotation. We are evaluating this functionality for the task of identifying cell lines, which might support our classification task, in addition to the machine learning approach.

For this purpose, we created the Cellosaurus_v33 dictionary (http://pubdictionaries.org/dictionaries/Cellosaurus_v33) that includes cell lines released in version 33 of Cellosaurus [3]. Based on this dictionary, we created a corresponding annotator in PubAnnotation (http://pubannotation.org/annotators/Cellosaurus_v33), which is a Web service that can be applied to any article in PubAnnotation for real-time annotation. We are currently developing a pre-processing script to filter out cell line names that match to a list of stopwords. Further, a post-processing script will also be applied to filter out mentions that match entities returned by PubTator Central, which are potential false positives.

## Conclusion

We presented the integration of the PubAnnotation ecosystem in our planned Web application which aims to mine alternative methods to animal experiments. We use all main functionalities of the ecosystem, namely, the PubAnnotation repository for the storage and alignment of annotations, PubDictionaries for dictionary matching of cell lines, and the TextAE annotation tool for the visualization of articles and annotations. Two repositories for annotations of discourse elements were created and are available to the research community. Further, these two repositories are being frequently and automatically updated by our Web application. Finally, we also released a cell line dictionary and its corresponding annotator.

## References

[1] Kim JD, Wang Y, Fujiwara T, Okuda S, Callahan TJ, Cohen KB (2019). Open Agile text mining for bioinformatics: the PubAnnotation ecosystem. Bioinformatics.

[2] Lin J, Wilbur WJ (2007). PubMed related articles: a probabilistic topic-based model for content similarity. BMC Bioinformatics.

[3] Bairoch A (2018). The Cellosaurus, a cell-line knowledge resource. J Biomol Tech.

[4] Mrabet Y, Kilicoglu H, Demner-Fushman D TextFlow: a text similarity measure based on continuous sequences.

[5] Neves M, Butzke D, Grune B Evaluation of scientific elements for text similarity in biomedical publications.

[6] Lauscher A, Glavas G, Eckert K ArguminSci: a tool for analyzing argumentation and rhetorical aspects in scientific writing.

[7] Wei CH, Allot A, Leaman R, Lu Z (2019). PubTator central: automated concept annotation for biomedical full text articles. Nucleic Acids Res.

